# Comprehensive analyses of PDHA1 that serves as a predictive biomarker for immunotherapy response in cancer

**DOI:** 10.3389/fphar.2022.947372

**Published:** 2022-08-08

**Authors:** Langmei Deng, Anqi Jiang, Hanqing Zeng, Xiaoji Peng, Liying Song

**Affiliations:** ^1^ Department of Emergency, The Third Xiangya Hospital, Central South University, Changsha, HN, China; ^2^ Department of Pharmacy, The Third Xiangya Hospital, Central South University, Changsha, HN, China; ^3^ Department of Pharmacy, Yueyang Hospital of Traditional Chinese Medicine, Yueyang, HN, China

**Keywords:** PDHA1, pan-cancer, immunotherapy, mutation, prognosis

## Abstract

Recent studies have proposed that pyruvate dehydrogenase E1 component subunit alpha (PDHA1), a cuproptosis-key gene, is crucial to the glucose metabolism reprogram of tumor cells. However, the functional roles and regulated mechanisms of PDHA1 in multiple cancers are largely unknown. The Cancer Genome Atlas (TCGA), GEPIA2, and cBioPortal databases were utilized to elucidate the function of PDHA1 in 33 tumor types. We found that PDHA1 was aberrantly expressed in most cancer types. Lung adenocarcinoma (LUAD) patients with high PDHA1 levels were significantly correlated with poor prognosis of overall survival (OS) and first progression (FP). Kidney renal clear cell carcinoma (KIRC) patients with low PDHA1 levels displayed poor OS and disease-free survival (DFS). However, for stomach adenocarcinoma (STAD), the downregulated PDHA1 expression predicted a good prognosis in patients. Moreover, we evaluated the mutation diversity of PDHA1 in cancers and their association with prognosis. We also analyzed the protein phosphorylation and DNA methylation of PDHA1 in various tumors. The PDHA1 expression was negatively correlated with tumor-infiltrating immune cells, such as myeloid dendritic cells (DCs), B cells, and T cells in pan-cancers. Mechanically, we used single-cell sequencing to discover that the PDHA1 expression had a close link with several cancer-associated signaling pathways, such as DNA damage, cell invasion, and angiogenesis. At last, we conducted a co-expressed enrichment analysis and showed that aberrantly expressed PDHA1 participated in the regulation of mitochondrial signaling pathways, including oxidative phosphorylation, cellular respiration, and electron transfer activity. In summary, PDHA1 could be a prognostic and immune-associated biomarker in multiple cancers.

## Introduction

The incidence and mortality of cancers are growing rapidly worldwide. The extremely complex process of tumorigenesis and poor prognosis is still a great challenge for cancer treatment (Zhang et al., 2017; Li C et al., 2020; Bray et al., 2020). Thus, it is urgent to explore novel candidate genes for making early diagnosis and predicting the prognosis in various malignancies. Pan-cancer analysis is highly significant and realizable for the evaluation of novel cancer-associated genes (ICGC/TCGA Pan-Cancer Analysis of Whole Genomes Consortium, 2020).

Pyruvate dehydrogenase E1 component subunit alpha (PDHA1), a critical component of a pyruvate dehydrogenase (PDH) complex (PDC), is indispensable in glucose metabolism and participates in oxidative phosphorylation and tricarboxylic acid cycle in mitochondria (Patel et al., 2014). The PDC activity was regulated by PDH kinases 4 (PDK1-4) at three independent serine (Ser, S) residues, S293, S300, and S232 (Kolobova et al., 2001). The inactivation of PDHA1 promotes tumor glycolysis by downregulating the PDC activity (Yu et al., 2017). In head and neck squamous cell carcinoma (HNSC) patients, high expression of PDK1 significantly promoted the phosphorylation of PDHA1 at Ser-232, resulting in the poor outcome (Golias et al., 2016). In gastric cancer, downregulated PDHA1 promoted cancer progression by increasing glycolysis (Liu et al., 2018). In addition, PDHA1 also played a critical role in cancer chemoresistance. In esophageal cancer KYSE450 cells, PDHA1 knockout could promote the resistance of docetaxel and paclitaxel through enhancing glycolysis (Liu et al., 2019). In prostate cancer, silencing PDHA1 significantly enhanced resistance to chemotherapy by inducing anaerobic glycolysis and enhancing migration ability (Li et al., 2016). Nevertheless, the detailed roles of PDHA1 in various cancers remain largely unclear.

In our study, a pan-cancer analysis was performed to explore the role and mechanism of PDHA1 in 33 human cancer types. By utilizing multiple bioinformatics tools, we carried out a systematic analysis of the prevalence and predictive values of PDHA1 in multiple tumor types. The altered characteristics of PDHA1 mainly contained its expression levels, mutation status, protein phosphorylation, and methylations. Furthermore, we explored the associations between PDHA1 expression and immunotherapy-associated signatures.

## Materials and methods

### Identification of PDHA1 expression based on bioinformatics databases

We compared the PDHA1 expression in tumor tissue and normal tissues by performing Tumor Immune Estimation Resource (TIMER2) (Li T et al., 2020) and Gene Expression Profiling Interactive Analysis (GEPIA2) (Tang et al., 2019). In GEPIA2, the *p*-value cutoff was 0.05, and the log2 (fold change) cutoff was 1. Next, we utilized the GEPIA2 database to analyze the association between PDHA1 expression and pathological stages in 33 cancer types. Using the Clinical Proteomic Tumor Analysis Consortium (CPTAC) (Edwards et al., 2015), we analyzed the protein expression, protein and phosphoprotein levels, and DNA methylation of PDHA1. Z-values represent standard deviations from the median across samples for the given cancer type. Log2 spectral count ratio values from CPTAC were first normalized within each sample profile and then normalized across samples. The information and the characteristics of the samples and cohorts from GEPIA2 are displayed in Supplementary Table S1.

### Survival prognosis analysis

Across 33 tumor types, the prognostic values of PDHA1, including overall survival (OS), first progression (FP), disease-free survival (DFS), and progression-free survival (PFS), were performed in the GEPIA2 database, The Cancer Genome Atlas (TCGA) (Wang et al., 2016), and the Kaplan–Meier plotter (Hou et al., 2017). The heatmap data and survival plots of PDHA1 were displayed. In addition, by using the cBioPortal tool (Gao et al., 2013), we explored the mutation frequency, mutation type, and site information of PDHA1 across 33 tumors. Also, we assessed the survival values of PDHA1 genetic alteration, including OS and DFS, across 33 cancers. The patients’ alteration information is displayed in Supplementary Table S2. The characteristics of PDHA1 mutation in samples and cohorts are displayed in Supplementary Table S3. The clinical information on PDHA1 alterations is displayed in Supplementary Table S4.

### Analysis of immune infiltration

We analyzed the relationship between PDHA1 expression and immune infiltrates across all tumors by using the TIMER2 tool. We selected B cell, natural killer cell (NK cell), macrophage cell, dendritic cell (DC), CD8+ T cell, neutrophil, monocyte cell, cancer-associated fibroblast (CAF), and regulatory T cells (Tregs) for detailed analysis. Seven algorithms, namely, TIMER, EPIC, MCPCOUNTER, CIBERSORT, CIBERSORT-ABS, QUANTISEQ, and XCELL, were applied for the analysis of immune infiltration.

### Analysis of single-cell sequencing data

At the single-cell level, we explored correlation data between PDHA1 expression and different tumor functional statuses by searching CancerSEA (Yuan et al., 2019). We drew a heatmap to indicate the significant correlation. The top four significantly different functional states (*p* < 0.0001) and the T-SNE diagram in tumors were obtained based on the CancerSEA database. The correlation and *p*-value of the cancer category and tumor functional status are displayed in Supplementary Table S5. The correlation matrix data are displayed in Supplementary Table S6.

### Enrichment analysis of PDHA1-related genes

The STRING website was utilized for the molecule interaction network analysis (Franceschini et al., 2013). Furthermore, GEPIA2 was used to download the top 100 similar genes of PDHA1 in pan-cancer (Supplementary Table S7). Next, using the Xiantao bioinformatics toolbox (https://www.xiantao.love/products), we explored the Pearson correlation between PDHA1 and the selected genes. In addition, a heatmap of the expression profile for the selected genes was obtained. GO and KEGG enrichment analyses about PDHA1 similar genes were performed by the Xiantao bioinformatics toolbox.

### Statistical analysis

In TIMER2, the statistical significance computed by the Wilcoxon test is annotated by the number of stars. In GEPIA2, we used the ANOVA method to compare tumor vs. all normal samples. We utilized Spearman’s rank correlation coefficient to evaluate the correlation between two groups. We used the Kaplan–Meier method to assess the association between prognosis of patients and PDHA1 expression or mutation levels. *p* < 0.05 was considered a statistically significant difference (Yang et al., 2020).

## Results

### Aberrant expression of PDHA1 in pan-cancer

**FIGURE 1:**
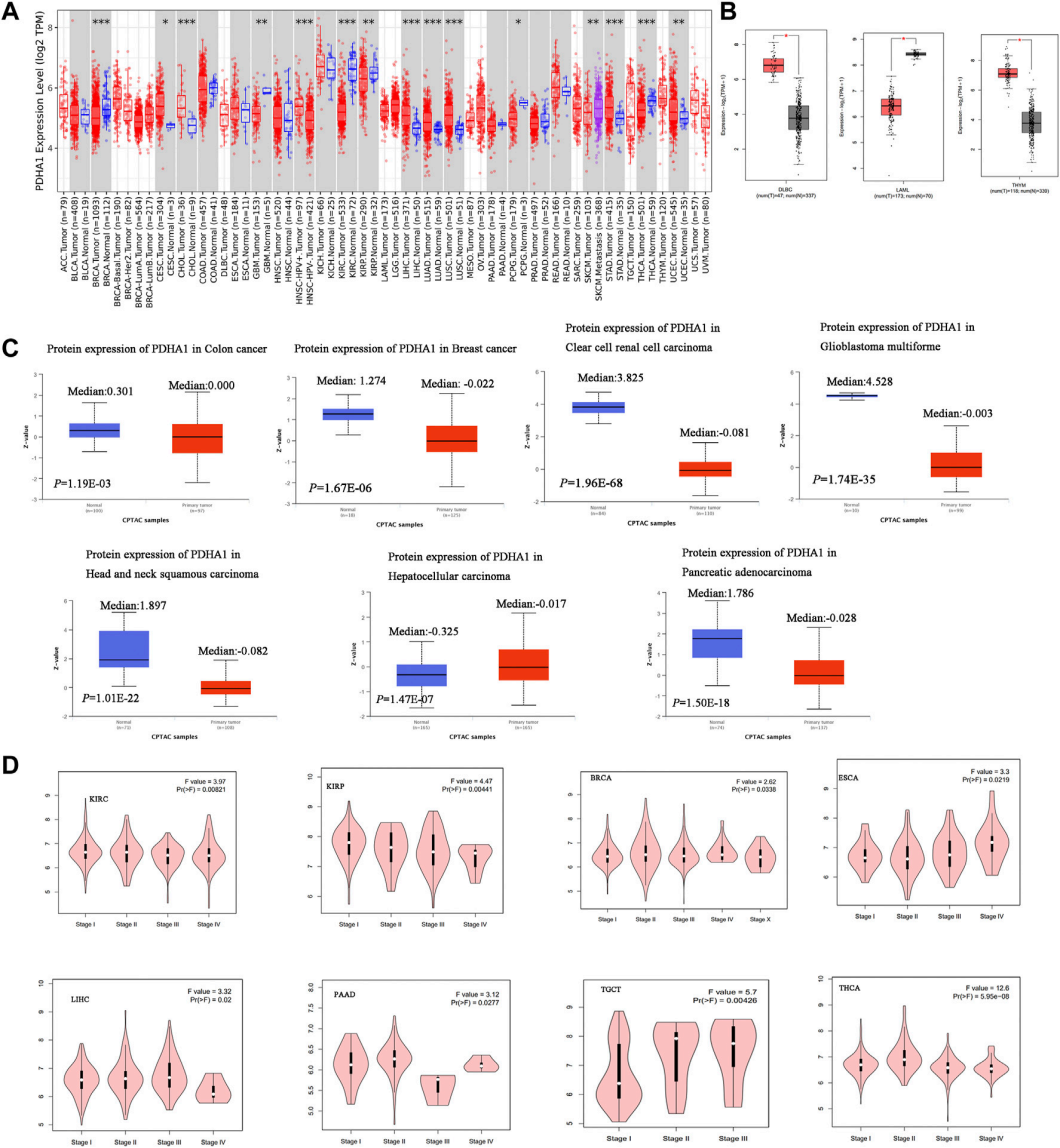
Aberrant expression of PDHA1 in pan-cancer. **(A)** mRNA level of PDHA1 performed by the TIMER2 database. **(B)** Box plot of PDHA1 mRNA level in DLBC, LAML, and THYM performed by the GEPIA2 database. **(C)** Total protein level of PDHA1 in normal tissue and COAD, BRCA, KIRC, GBM, HNSC, PAAD, and LIHC performed by CPTAC. **(D)** Relationship between PDHA1 expression and tumor pathological stage performed in GEPIA2. *p < 0.05; **p < 0.01; ***p < 0.001.

In this study, TIMER2 was used to research the differential expression of PDHA1 by comparing tumors and normal tissues. As shown in Figure 1A, the PDHA1 expression in seven tumor tissues, namely, cervical squamous cell carcinoma and endocervical adenocarcinoma (CESC), cholangiocarcinoma (CHOL), liver hepatocellular carcinoma (LIHC), lung adenocarcinoma (LUAD), lung squamous cell carcinoma (LUSC), stomach adenocarcinoma (STAD), and uterine corpus endometrial carcinoma (UCEC), was significantly upregulated. In contrast, PDHA1 was significantly downregulated in six tumors, namely, breast invasive carcinoma (BRCA), glioblastoma multiforme (GBM), kidney renal clear cell carcinoma (KIRC), kidney renal papillary cell carcinoma (KIRP), pheochromocytoma and paraganglioma (PCPG), and thyroid carcinoma (THCA). We further assessed the differential expression of PDHA1 between tumor and normal tissues by matching TCGA and GTEx in several cancers. We found the upregulated expression of PDHA1 in lymphoid neoplasm diffuse large B-cell lymphoma (DLBC) and thymoma (THYM) and the downregulated expression of PDHA1 in acute myeloid leukemia (LAML) (Figure 1B). For other tumors, there were no significant differences in the expression of PDHA1 (Supplementary Figure S1A).

To better understand the differential expression, the CPTAC dataset was used to assess the PDHA1 protein level in large-scale proteome data from the National Cancer Institute. As shown in Figure 1C, the total protein expression of PDHA1 was significantly decreased in colon adenocarcinoma (COAD), BRCA, KIRC, GBM, HNSC, and pancreatic adenocarcinoma (PAAD) and elevated in LIHC. The total protein expression of PDHA1 in LUAD, ovarian serous (OV), and UCEC showed no differential expression (Supplementary Figure S1B).

The GEPIA2 tool was also used to analyze the relationship between the PDHA1 expression and tumor pathological stage. Figure 1D showed stage-specific change of PDHA1 in eight tumor types, including KIRC, KIRP, BRCA, THCA, PAAD, testicular germ cell tumors (TGCTs), and esophageal carcinoma (ESCA). In other cancers, there was no clear association between the PDHA1 expression and patients’ stage (Supplementary Figure S1C).

### Survival analysis of PDHA1 expression in pan-cancer

**FIGURE 2:**
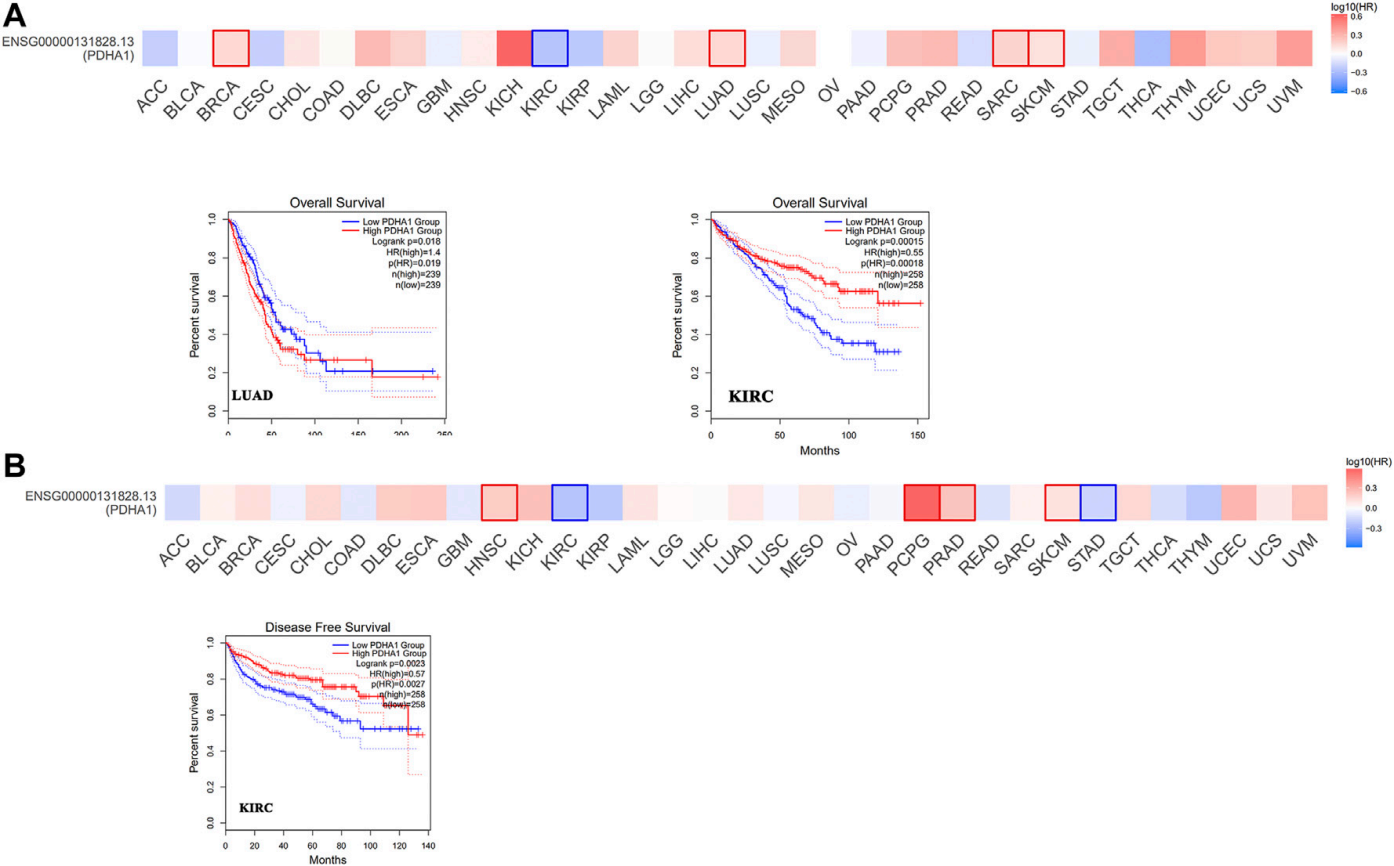
Survival analysis of PDHA1 expression in pan-cancer. **(A,B)** GEPIA2 indicated the roles of PDHA1 expression in patients’ prognosis, including OS **(A)** and DFS **(B)**. High cutoff (50%) and low cutoff (50%) values were used as the expression thresholds for splitting the high-expression and low-expression cohorts. The cutoff value was as follows: *p*-value < 0.01 and | logFC| > 1.

Next, we used GEPIA2 to explore the role of PDHA1 in patients’ prognosis, including OS and DFS. High expression of PDHA1 was associated with poor prognosis in patients with LUAD (*p* = 0.019). Inversely, high expression of PDHA1 was associated with good prognosis in patients with KIRC (Figures 2A,B). Furthermore, we used the Kaplan–Meier plotter tool to identify the survival values of PDHA1. As shown in Supplementary Figure S2, we found that a high PDHA1 expression level was associated with poor prognosis in patients with lung cancer and STAD. These results indicated the promising roles of PDHA1 in the patients’ prognosis of lung cancer.

### Genetic alteration of PDHA1 in pan-cancer

**FIGURE 3:**
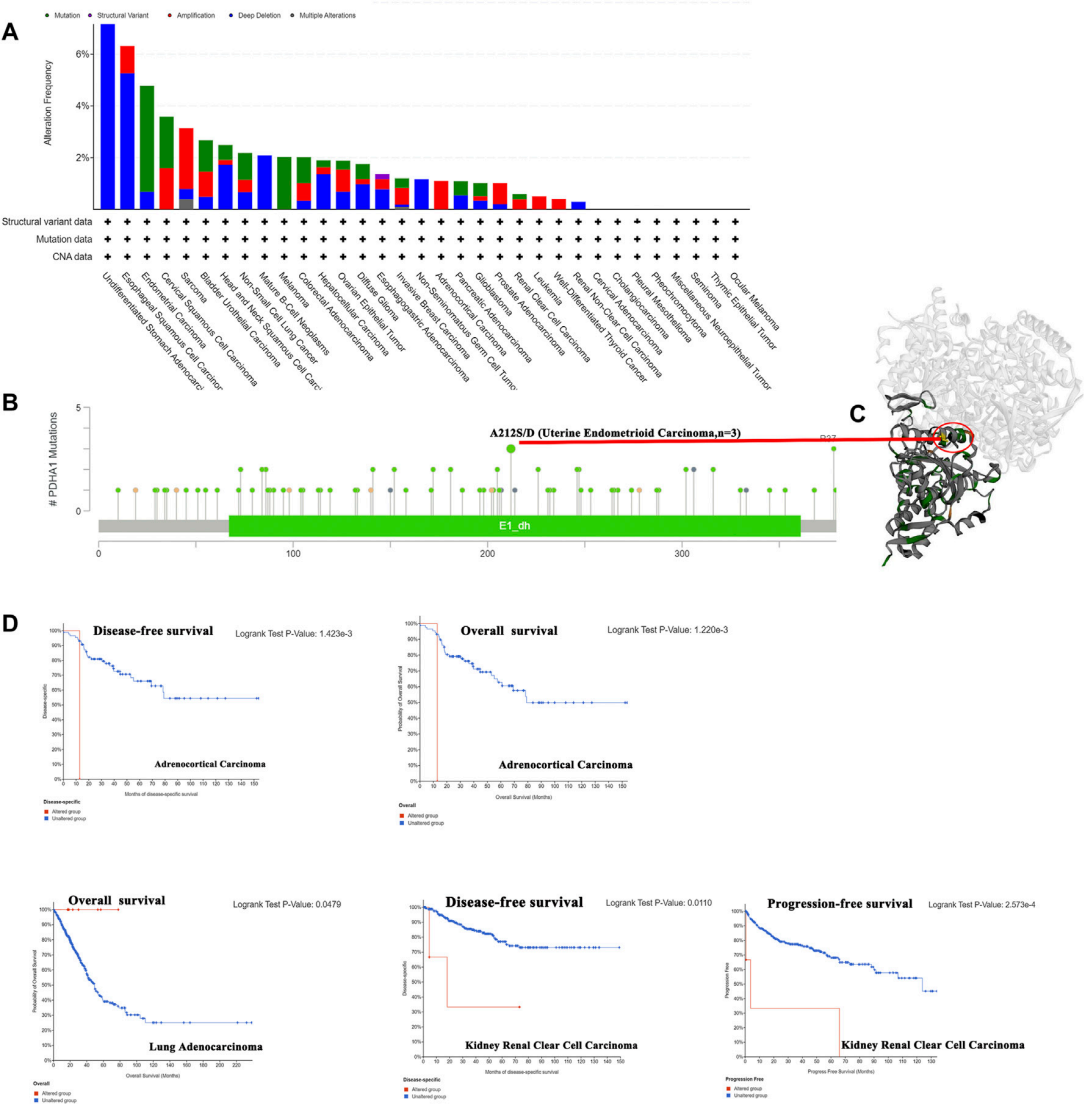
Genetic alteration of PDHA1 in pan-cancer. **(A)** Mutation status of PDHA1 in pan-cancers was performed by the cBioPortal tool. **(B)** Main mutation types of PDHA1. **(C)** A212S/D mutation site was visualized in the 3D structure of PDHA1 protein. **(D)** Roles of PDHA1 alteration in the patients’ prognosis, including OS, DFS, and PFS.

Genetic alterations have been proved to affect tumorigenesis and treatment (Yang Z et al., 2021). Thus, we explored the PDHA1 genetic alterations in human tumor samples. According to our analysis, the frequency of PDHA1 alteration (7.69%) is the highest in undifferentiated STAD with “deep deletion” as the primary type. Endometrial carcinoma had the highest incidence of the “mutation” type with a frequency of 4.1% (Figure 3A). As shown in Figure 3B, there were 92 mutations in the full sequence of PDHA1. Also, “mutation” seemed to be the main type of genetic alteration, which is mainly located within the dehydrogenase E1 component (E1_dh) domain (67–361). For instance, a missense mutation with potential clinical significance, A212S/D alteration, was only detected in three cases of uterine endometrioid carcinoma. Also, the A212S/D site was visualized in the 3D structure of PDHA1 protein (Figure 3C). After this, we systematically explored the relationship between genetic alterations of PDHA1 and the clinical survival prognosis of patients. As shown in Figure 3D, the genetic alteration of PDHA1 showed a poor prognosis in adrenocortical carcinoma (ACC) and KIRC patients and a good prognosis in LUAD patients.

### Protein phosphorylation and DNA methylation of PDHA1 in pan-cancer

**FIGURE 4:**
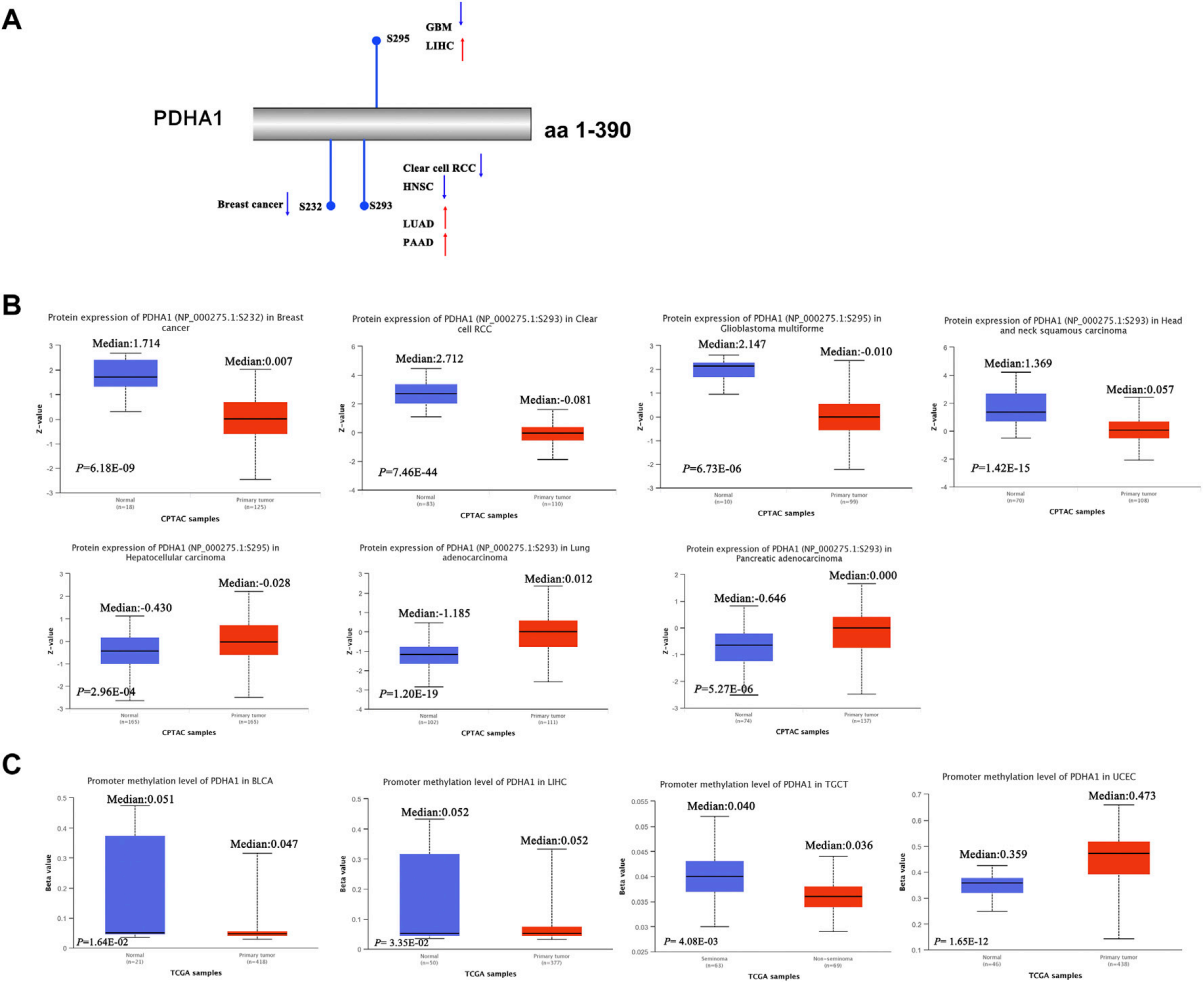
Protein phosphorylation and DNA methylation of PDHA1 in pan-cancer. **(A,B)** CPTAC indicated the phosphorylation levels of PDHA1 at S232, S293, and S295. **(C)** DNA methylation of PDHA1 between normal and primary tumor tissues was performed by the UALCAN database.

Recent studies have demonstrated that PDHA1 phosphorylation could promote tumor migration ability and therapeutic resistance by suppressing its PDH activity (Zimmer et al., 2016; Jin et al., 2021). We further explored the phosphorylation of PDHA1 between normal and primary tumor tissues. Using the CPTAC dataset, we found the decreased phosphorylation level of S232 for BRCA, decreased phosphorylation level of S293 for KIRC and HNSC, increased phosphorylation level of S293 for LUAD and PAAD, decreased phosphorylation level of S295 for GBM, and increased phosphorylation level of S295 for LIHC (Figures 4A,B).

In addition, in multiple cancers, DNA methylation throughout the genome is an epigenetic modification contributing to the regulation of cancer-associated genes (Liu D et al., 2021; Rogozin et al., 2021). Research studies have verified that PDK4 methylation could display the oncogenic roles in colon cancer (Leclerc et al., 2017). However, the underlying roles of PDHA1 methylation in various cancers remain largely unclear. In our study, we demonstrated the decreased promoter methylation level of PDHA1 for BLCA, LIHC, and TGCT and increased promoter methylation level of PDHA1 for UCEC (Figure 4C). No obvious changes in methylation values of PDHA1 could be found in other cancers (Supplementary Figure S3).

### The roles of PDHA1 in the immune infiltration in pan-cancer

**FIGURE 5:**
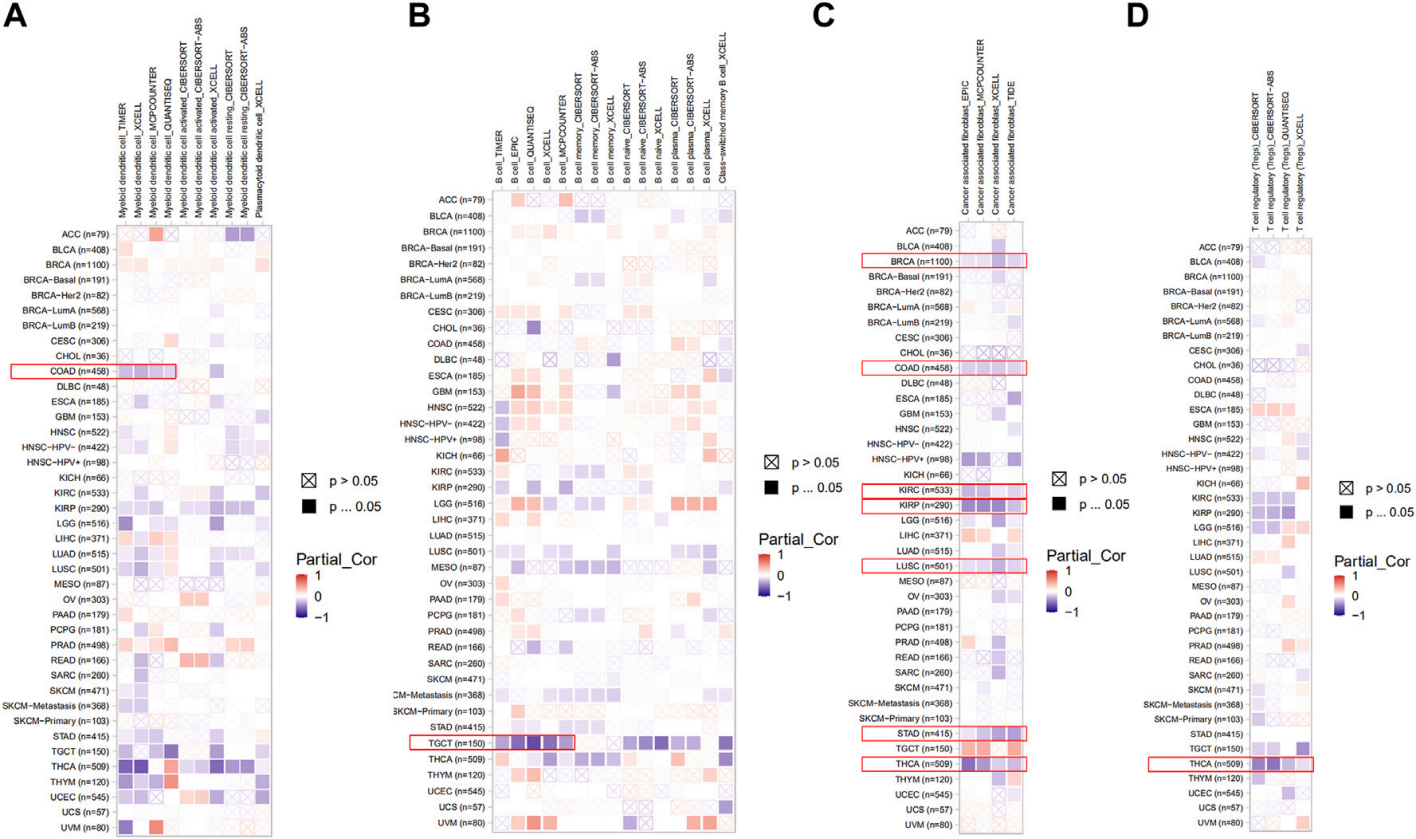
Roles of PDHA1 in the immune infiltration in all TCGA tumor types. **(A–D)** Correlation heatmap between PDHA1 expression and tumor-infiltrating immune cells across 33 cancer types was displayed, including DC **(A)**, B cell **(B)**, CAF **(C)**, and Tregs **(D)**. A positive correlation was marked as red color, while a negative correlation was marked as blue color. Nonsignificant correlations values were marked with a cross.

Here, we explored the potential correlation between PDHA1 expression and tumor-infiltrating immune cells by performing comprehensive research. Seven algorithms, namely, TIMER, EPIC, MCPCOUNTER, CIBERSORT, CIBERSORT-ABS, QUANTISEQ, and XCELL, were applied for the estimation of immune infiltration cells in all TCGA tumor types. As shown in Figures 5A–D, we discovered a negative correlation between the PDHA1 expression and the estimated infiltration value of myeloid DC for COAD, B cell for TGCT, T cell for THCA, and CAF for BRCA, COAD, KIRC, KIRP, LUSC, STAD, and THCA. There was no significant correlation between PDHA1 levels and other tumor-infiltrating immune cells, such as NK cell, macrophage, neutrophil, monocyte cell, and Tregs (Supplementary Figure S4).

### Expression pattern of PDHA1 in a single cell and its relationship with the cancer functional status

**FIGURE 6:**
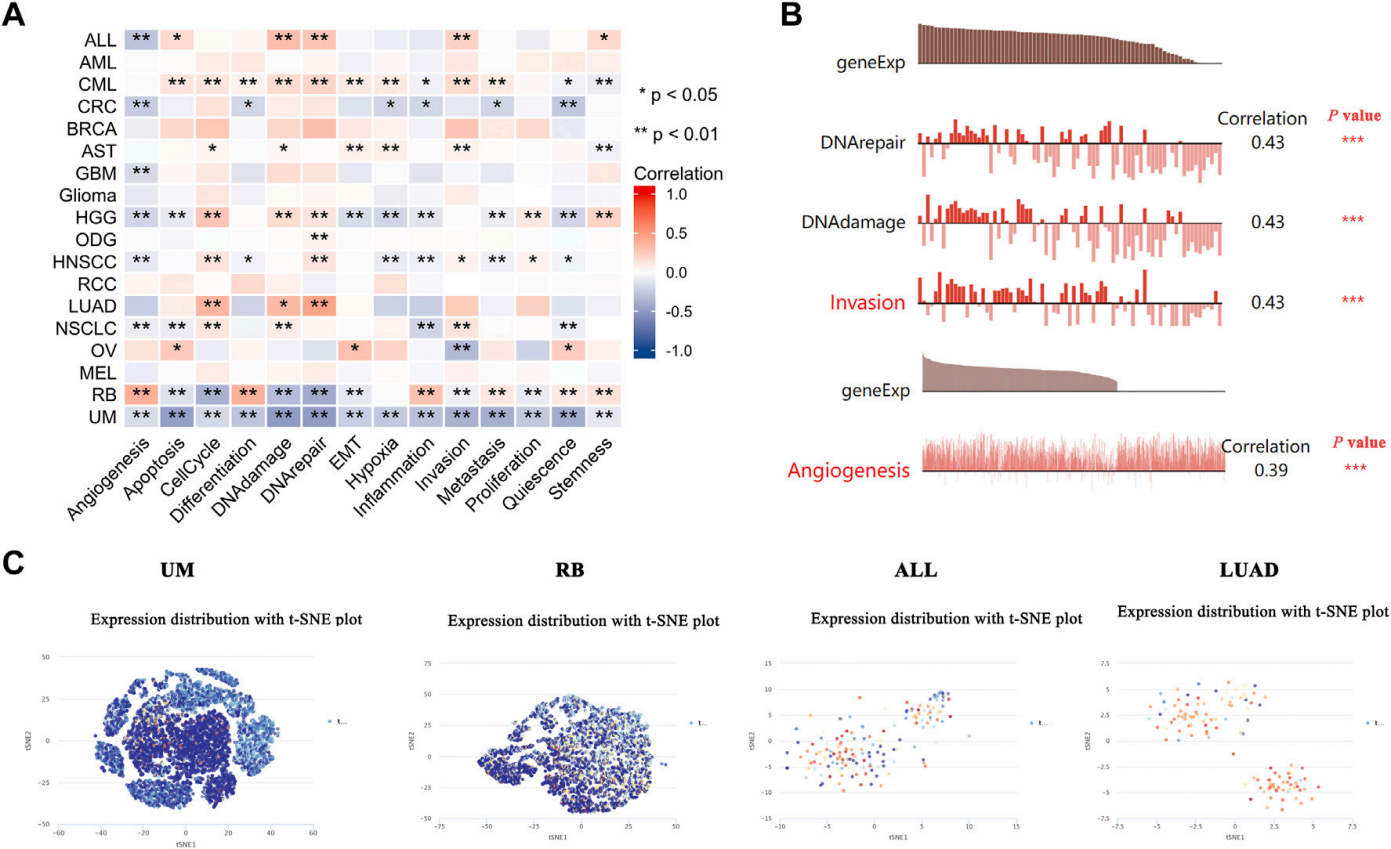
Expression pattern of PDHA1 at the single-cell level and its relationship with the cancer functional status. **(A)** Correlation between PDHA1 expression and different tumor functional status was displayed as a heatmap performed by the CancerSEA database. **(B)** Correlation between PDHA1 expression and four significantly different functional states. **(C)** PDHA1 expression profiles at single-cell levels of UM, RB, ALL, and LUAD by the T-SNE diagram. *p < 0.05; **p < 0.01; ***p < 0.001.

The recently developed single-cell sequencing technologies could be used to overcome the cell heterogeneity in tumors (Liu J et al., 2021). We searched the CancerSEA website to verify the expression of PDHA1 at the single-cell level in different cancers and its relationship with the tumor functional status. As shown in Figure 6A, the heatmap showed that PDHA1 had a strong correlation with fourteen tumor functional statuses in most cancer types. Figure 6B showed the positive relationship between the PDHA1 expression and DNA repair in uveal melanoma (UM), DNA damage in retinoblastoma (RB), invasion in acute lymphoblastic leukemia (ALL), and angiogenesis in LUAD. PDHA1 expression profiles were shown at single-cell levels of UM, RB, ALL, and LUAD by a T-SNE diagram (Figure 6C). The scatter plot describing the correlations between the gene expression and tumor functional status was added as a supplement (Supplementary Figure S5). These results suggested that PDHA1 might play a crucial role in the biological processes of cancer progression.

### Co-expression network of PDHA1 and enrichment pathway analysis

**FIGURE 7:**
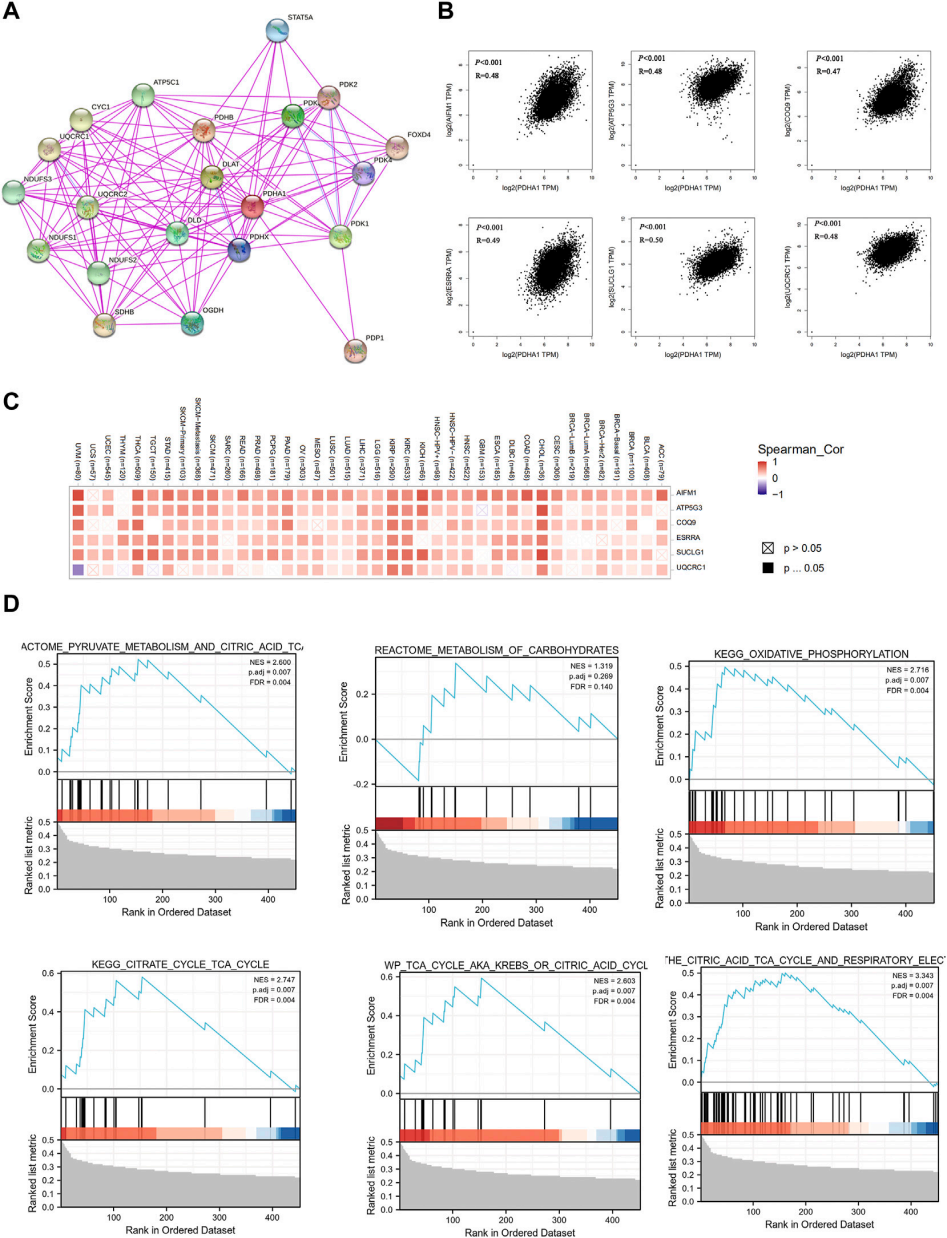
Co-expression network and enrichment pathway analysis of PDHA1. **(A)** In total, 20 experimentally identified PDHA1-binding molecules were acquired by STRING. **(B)** Top six PDHA1-correlated genes analyzed by GEPIA2, including *AIFM1*, *ATP5G3*, *COQ9*, *ESRRA*, *SUCLG1*, and *UQCRC1*. **(C)** Heatmap representation of the expression correlation between PDHA1 and the top six PDHA1-correlated genes, *AIFM1*, *ATP5G3*, *COQ9*, *ESRRA*, *SUCLG1*, and *UQCRC1*, in 33 tumors. **(D)** Roles of PDHA1 in the metabolism signaling pathway by performing GSEA.

Finally, to better understand the molecular mechanism of PDHA1 in tumorigenesis and development, we used the STRING tool to construct the PDHA1-interacted molecule network. A total of 20 experimentally identified PDHA1-binding molecules were acquired (Figure 7A). Then, we used the GEPIA2 tool to acquire the top 100 similar genes correlated with PDHA1 expression. The expression of PDHA1 was positively associated with apoptosis-inducing factor mitochondria associated 1 (AIFM1, R = 0.48), ATP synthase membrane subunit C locus 3 (ATP5G3, R = 0.48), coenzyme Q9 (COQ9, R = 0.47), estrogen-related receptor alpha (ESRRA, R = 0.49), succinate-CoA ligase GDP/ADP-forming subunit alpha (SUCLG1, R = 0.5), and ubiquinol-cytochrome C reductase core protein 1 (UQCRC1, R = 0.48) (Figure 7B and Supplementary Table S8). The heatmap demonstrated that PDHA1 had a strong positive correlation with the six aforementioned genes in most cancer types (Figure 7C). By performing GSEA, we further verified the potential roles of PDHA1-associated molecules in the regulation of metabolism signaling pathways, including pyruvate metabolism, metabolism of carbohydrates, oxidative phosphorylation, and citric acid cycle (Figure 7D). In addition,Supplementary Figure S6A and Supplementary Table S9 showed that PDHA1 was involved in tumorigenesis through the regulation of oxidative phosphorylation, Parkinson’s disease, nonalcoholic fatty liver disease, and thermogenesis by performing KEGG analysis. GO enrichment also indicated that PDHA1-associated molecules were significantly related to cellular respiration, electron transfer activity, and mitochondrial inner membrane (Supplementary Figures S6B–D; Supplementary Table S9).

## Discussion

PDHA1, a key component of PDH, is a rate-limiting enzyme complex for maintaining the tricarboxylic acid cycle (TCA cycle). Emerging studies have demonstrated that cancer metabolism regulated by PDHA1 plays a key role in cancer progression and metastasis (Yetkin-Arik et al., 2019). Tsvetkov et al. (2022) demonstrated that copper-induced cell death is a novel cell death involved in human tumors. Using a whole-genome CRIPSR-Cas9 positive selection screen, they found that PDHA1 may play a pivotal role in malignancies by regulating cuproptosis. Nevertheless, the detailed role of PDHA1 in cancers and the underlying mechanism driving tumor pathogenesis are still largely unclear. Thus, we performed a pan-cancer analysis for PDHA1.

In our result, augmented levels of PDHA1 were observed in the tumor tissues of CESC, CHOL, LIHC, LUAD, LUSC, STAD, and UCEC, whereas low expression of PDHA1 was observed in BRCA, GBM, KIRC, KIRP, PCPG, and THCA. These results indicated that PDHA1 may play different roles in different types of cancers. In addition, we verified that upregulated PDHA1 predicted poor OS for patients in LUAD, good OS and DFS for patients in KIRC, and poor DFS for patients in KIRC. The Kaplan–Meier plotter identified that a high PDHA1 expression level was associated with poor prognosis of OS and PPS for STAD. This suggested that PDHA1 may be a potential biomarker for predicting the prognosis of tumor patients.

In lung cancer, the study of Cevatemre et al. (2021) showed that knockdown of PDHA1 expression confers chemoresistance in A549 cells by inducing the epithelial–mesenchymal transition process. The study of Ma et al. (2018) demonstrated that dichloroacetate acid (DCA), a pyruvate dehydrogenase kinase inhibitor, could produce a therapeutic benefit in A549 and H1299 cells by activating PDHA1. Here, in LUAD, we demonstrated that patients with PDHA1 genetic alteration have a better prognosis in OS. Also, the phosphorylation level of PDHA1 S293 increased in LUAD. The single-cell transcriptomic sequencing study suggested that PDHA1 expression was significantly associated with several cancer-associated signaling in LUAD, including cell cycle, DNA damage, and DNA repair.

Previous research has demonstrated that decreased SIRT5 expression in KIRC accelerated the Warburg effect through PDHA1 hypersuccinylation, resulting in tumorigenesis and progression (Yihan et al., 2021). Here, in KIRC, we found a negative correlation between the expression of PDHA1 and clinical staging by exploring TCGA-KIRC datasets. Furthermore, the phosphorylation level of PDHA1 S293 significantly decreased in KIRC. Patients with PDHA1 genetic alteration displayed a poor prognosis in DFS and PFS. These results indicated that aberrant alterations of PDHA1 might participate in the KIRC progression and prognosis.

Currently, the tumor microenvironment (TME) makes a significant impact on malignancies (Bi et al., 2020; Jia et al., 2021; Li et al., 2021). As a major component of TME, CAF has multiple pro-tumorigenic functions during tumorigenesis (Yang W et al., 2021). Sun et al. (2019) demonstrated that IL-6 was increased in the supernatant of isolated CAFs, which could promote BRCA cell proliferation. In recent years, emerging studies have proved the well-established role of B cells in shaping antitumor immunity. Song et al. (2022) found that B-cell marker genes could effectively indicate the patients’ survival and provide targets for immunotherapy in lung cancer. Our study showed that the PDHA1 expression was negatively correlated with CAFs, DCs, B cells, and T cells in many cancers. However, the potential mechanism of PDHA1 in regulating TME requires further study.

Nevertheless, this study still has some limitations. First, the specific molecular mechanisms of PDHA1 on cuproptosis in multiple cancers have not been explored in this study, especially the roles of PDHA1 expression, genetic alterations, protein phosphorylation, and DNA methylation in the regulation of cuproptosis in tumor progression need to be further identified. Second, more *in vivo* and *in vitro* studies about the underlying mechanisms of PDHA1 in cancer progression require further investigation. Third, we found the double-edged roles of PDHA1 as oncogenes or tumor suppressors in different cancers, which might be due to the different origins of cancer cells and the tumor heterogeneity.

In summary, by performing a comprehensive pan-cancer analysis of PDHA1, we displayed the abnormal expression profiles of PDHA1 and its correlation with clinical prognosis and immune response. In addition, we also analyzed the protein phosphorylation and methylation values of PDHA1 in a variety of human cancers. These results could help to clarify the underlying functions of PDHA1 in tumorigenesis.

## Data Availability

The datasets presented in this study can be found in online repositories. The names of the repository/repositories and accession number(s) can be found in the article/Supplementary Material.

## Supplementary material

SUPPLEMENTARY FIGURE S1 Aberrant expression of PDHA1 in pan-cancer. **(A)** Box plot of the PDHA1 mRNA level analyzed by the GEPIA2 database. **(B)** Tumors with no significant differences in the protein level of PDHA1. **(C)** Tumors with no clear association between PDHA1 expression and patients’ stage.

SUPPLEMENTARY FIGURE S2 Survival analysis of PDHA1 expression in lung cancer and STAD. The Kaplan–Meier plotter indicated the roles of PDHA1 in patients’ prognosis in lung cancer and STAD, including FP, OS, and PPS.

SUPPLEMENTARY FIGURE S3 Tumors with no obvious changes in methylation values of PDHA1.

SUPPLEMENTARY FIGURE S4 Correlation between PDHA1 levels and other tumor-infiltrating immune cells, such as NK cell, macrophage, neutrophil, monocyte cell, and CD8+ T cells.

SUPPLEMENTARY FIGURE S5 Scatter plot of the correlations between PDHA1 gene expression and tumor functional status in LUAD and RB.

SUPPLEMENTARY FIGURE S6 KEGG and GO enrichment analyses about PDHA1 similar genes. **(A–D)** Roles of PDHA1 in the regulation of signaling pathways by performing GO and KEGG enrichment analyses.

SUPPLEMENTARY TABLE S1 Characteristics of samples and cohorts from the GEPIA2 database.

SUPPLEMENTARY TABLE S2 Alteration information and cancer study styles from the cBioPortal tool.

SUPPLEMENTARY TABLE S3 Information of PDHA1 mutation samples and cohorts from the cBioPortal tool.

SUPPLEMENTARY TABLE S4 Clinical information of PDHA1 alterations from the cBioPortal tool.

SUPPLEMENTARY TABLE S5 Correlation and p-value of 18 cancers and the tumor functional status from the CancerSEA database.

SUPPLEMENTARY TABLE S6 Correlation matrix data of 18 cancers from the CancerSEA database.

SUPPLEMENTARY TABLE S7 Top 100 similar genes of PDHA1 in pan-cancer from the GEPIA2 database.

SUPPLEMENTARY TABLE S8 Top six PDHA1-correlated genes in pan-cancer from the GEPIA2 database

SUPPLEMENTARY TABLE S9 GO/KEGG enrichment pathway analysis data about PDHA1 similar genes from the Xiantao bioinformatics toolbox.
