# Supplementary figures and images for: Comprehensive analyses of PDHA1 that serves as a predictive biomarker for immunotherapy response in cancer
Source: Front Pharmacol. 2022 Aug 8;13:947372. doi: 10.3389/fphar.2022.947372 (PMC9393251; doi:10.3389/fphar.2022.947372)

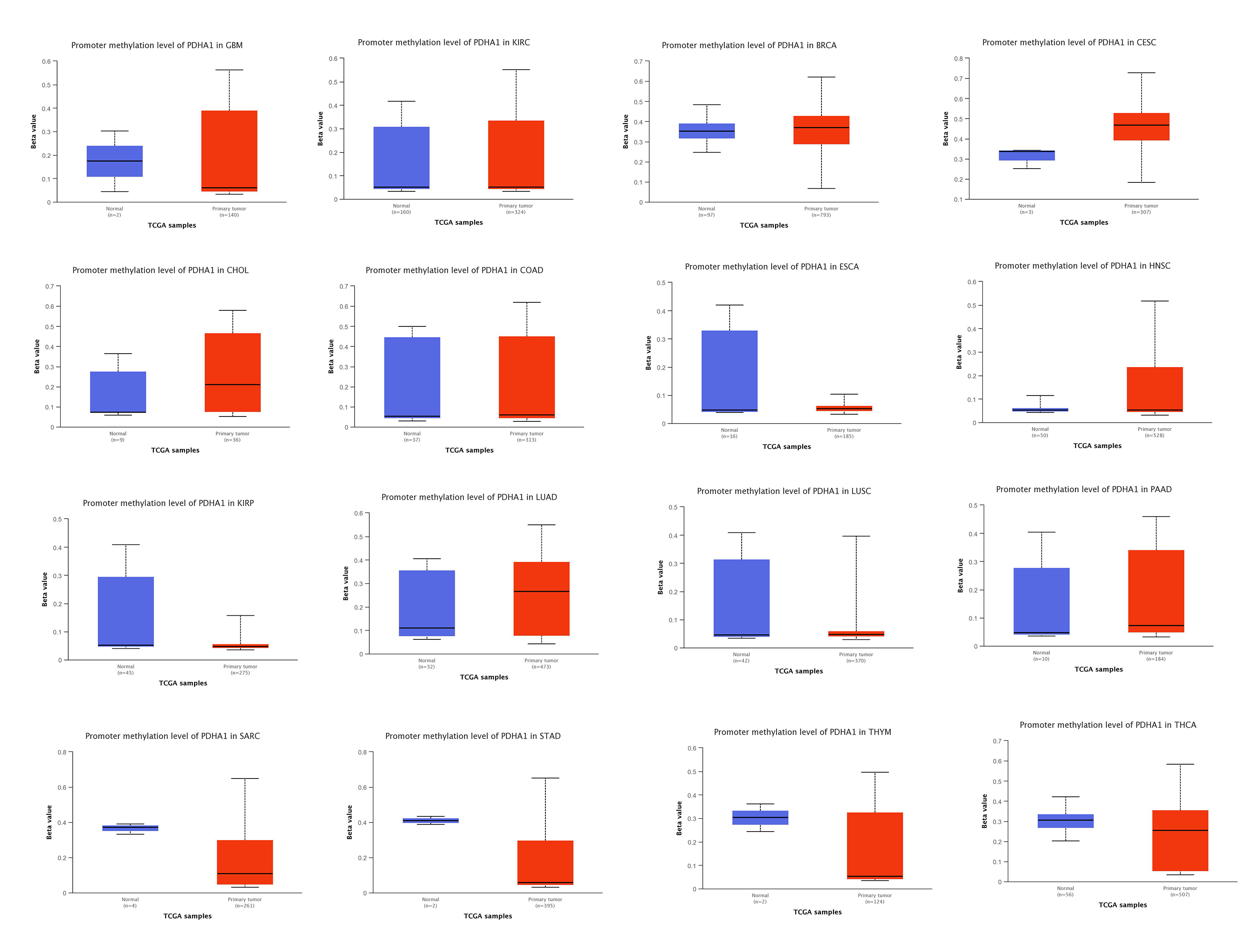

Supplement: Supplementary file 4 [file image3.tif]

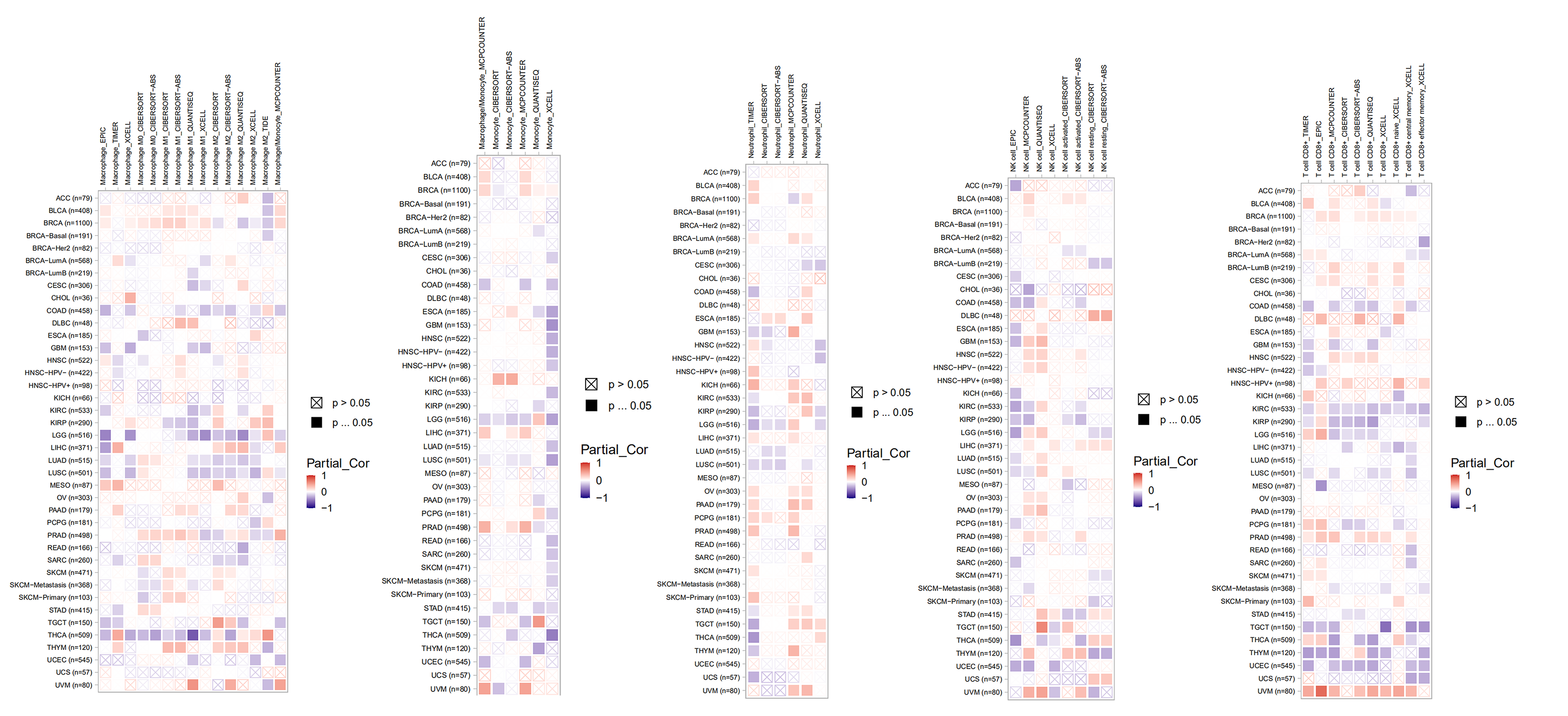

Supplement: Supplementary file 5 [file image4.tif]

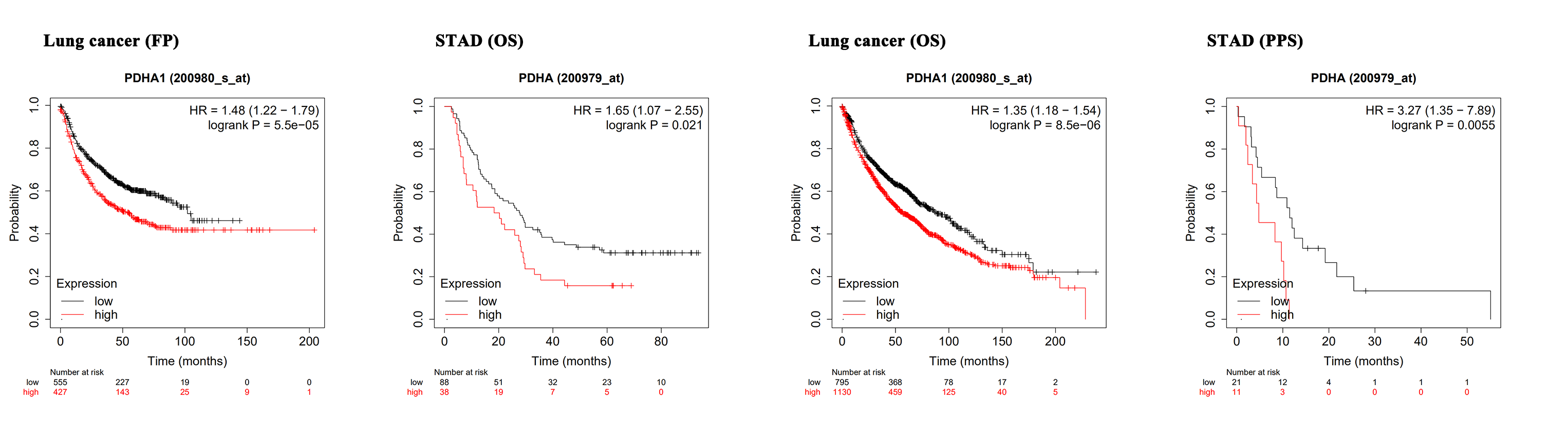

Supplement: Supplementary file 6 [file image2.tif]

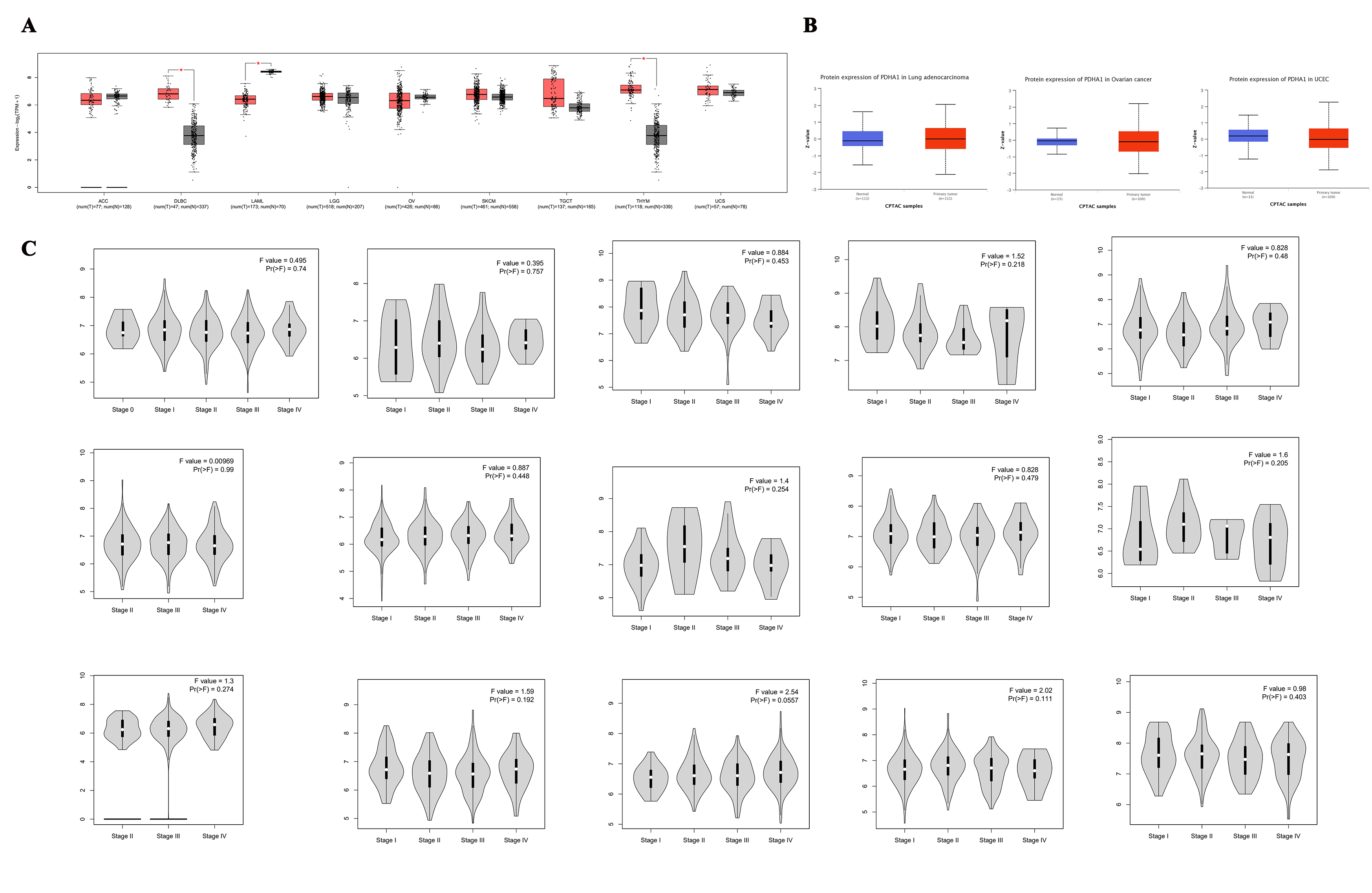

Supplement: Supplementary file 7 [file image1.tif]

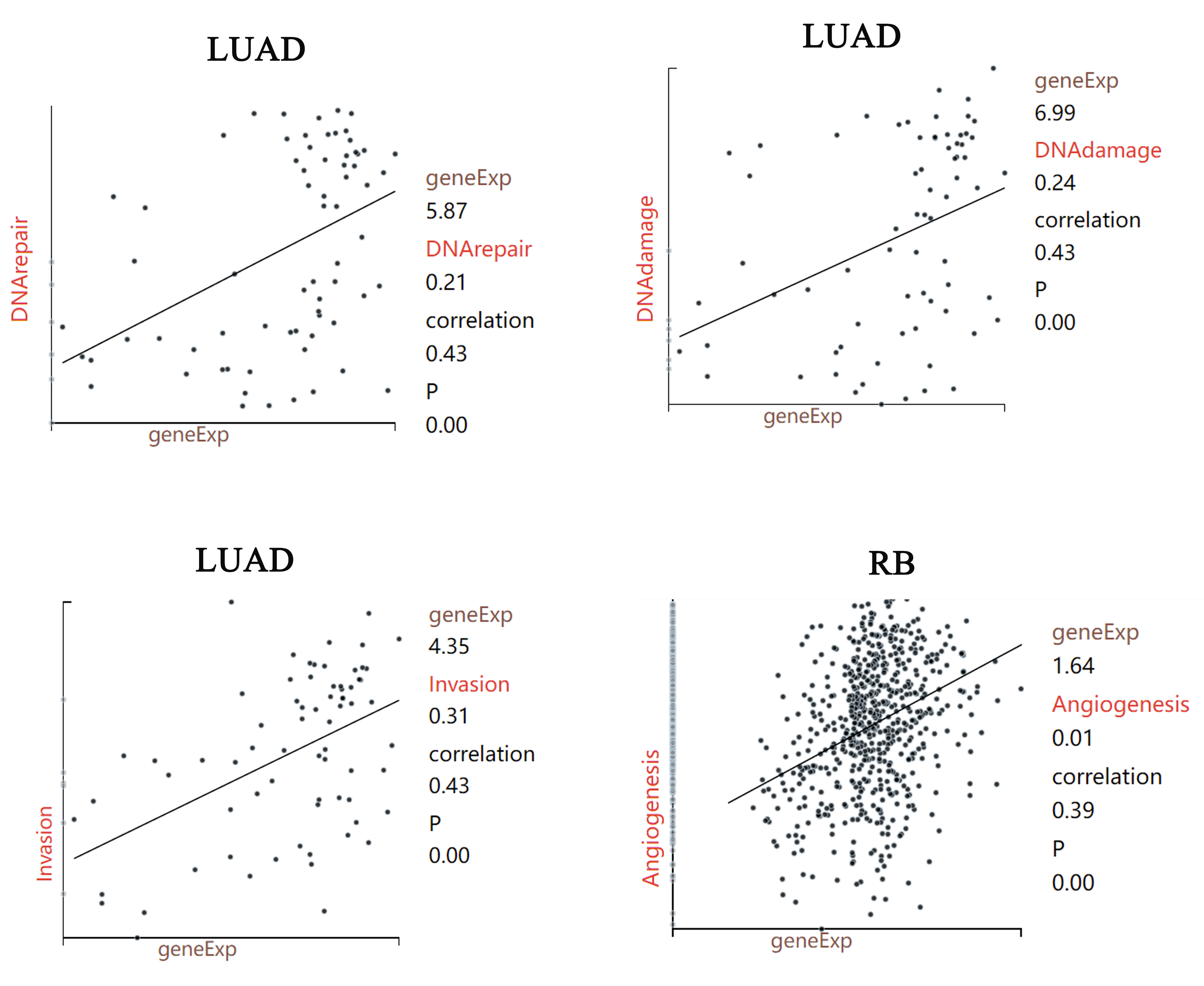

Supplement: Supplementary file 11 [file image5.tif]
